# Zinc oxide nanoparticles induce dose-dependent toxicosis in broiler chickens reared in summer season

**DOI:** 10.1007/s11356-022-19156-4

**Published:** 2022-03-16

**Authors:** Waleed M. Dosoky, Aya A. Al-Banna, Soliman M. Zahran, Soha A. Farag, Nader R. Abdelsalam, Asmaa F. Khafaga

**Affiliations:** 1 Department of Animal and Fish Production, Faculty of Agriculture (Saba Basha), Alexandria University, Alexandria, 21531 Egypt; 2 Department of Animal Production, Faculty of Agriculture, Tanta University, Tanta, Egypt; 3 Agricultural Botany Department, Faculty of Agriculture (Saba Basha), Alexandria University, Alexandria, 21531 Egypt; 4 Department of Pathology, Faculty of Veterinary Medicine, Alexandria University, Edfina, 22758 Egypt

**Keywords:** Zinc oxide nanoparticles, Histopathology, Immunity, Interleukin-6, Tumor necrosis factor, Growth performance

## Abstract

This research evaluates the effect of dietary zinc oxide nanoparticles’ (ZnO NPs) supplementation on growth performance, immunity, oxidative antioxidative properties, and histopathological picture of broiler chicken reared in the summer season. A total of 224 1-day-old male Cobb chicks were randomly allocated to seven groups of dietary treatments (*n* = 32). Seven isocaloric and isonitrogenous diets were formulated. ZnO NPs were added to the basal diet at seven different levels, 0, 5, 10, 20, 40, 60, and 80 ppm/kg diet, respectively, for 35 days. Results indicated that live body weight (g) did not differ significantly (*P* > *0.05*) between treatment groups, whereas compared to control, the 5 ppm ZnO NPs/kg diet recorded the highest live body weight at 21 and 35 days. No significant effects for the feed consumption (g/bird/period) and feed conversion ratio (g feed/g gain) among treated and control birds were observed. Hematological and immunological variables showed significant (*P* ≤ *0.05*) dose-dependent modulations by ZnO NP supplementation. Significant (*P* ≤ *0.05*) differences were observed in the phagocytic activity, phagocytic index, and IgM and IgG between the treatment groups, with the 5 and 10 ppm ZnO NPs/kg diet recording the best values, followed by the 20 ppm ZnO NPs/kg diet. Different supplementations had nonsignificant effects on the digestibility of nutrients (*P* ≤ *0.05*). Histopathological pictures of the kidney, liver, and lymphoid organs, ultrastructural examination of muscle tissues, and expression of inflammatory cytokines showed dose-dependent morphological and structural changes. In conclusion, the ZnO NP supplementation in broiler diet to eliminate the heat stress hazards in summer season is recommended in dose level of not more than 10 ppm/kg diet.

## Introduction

In tropical regions, high ambient temperature is considered one of the crucial factors inducing stress in birds. Due to worldwide warming, high temperature has lately become one of the greatest important stressors influencing the poultry industry (Jadhao et al. 2020; Lara and Rostagno 2013). Once the chicks are subjected to constant high temperatures, this exposure to heat negatively affects the performance and specific immune response and can lead to death, causing a large economic loss in the poultry industry (Abdel-Latif et al. 2018; Rao et al. 2016; Rossi et al. 2007).

The immune response and the antioxidant system of poultry can be enhanced using several feed additives, especially during heat stress, such as trace minerals, vitamins, and probiotics (Abdel-Latif et al. 2018; Dawood et al. 2019a, 2019b; Saeed et al. 2020; Zahin et al. 2020). Recently, more attention has been paid to adding zinc (Zn) supplementation in bird feed to improve the growth performance in broilers. This additive is increasingly required in bird diets during heat stress (Chand et al. 2021; Chand et al. 2020; De Grande et al. 2020; Hafez et al. 2020; Naz et al. 2016; Sahin et al. 2003; Shah et al. 2020) as it positively impacts the performance of chicken subjected to thermal challenges (Rao et al. 2016), enhancing the physiological responses, performance parameters, and immune response of heat-stressed birds (Shah et al. 2020). Moreover, it was reported that Zn and probiotics clearly modulated the intestinal microstructures of birds reared under high temperatures.

Zn is an essential trace mineral and vital for poultry’s metabolic functions, growth, and glandular improvement. It is a cofactor for the activity of up to 300 enzymes in birds (Salim et al. 2008). Moreover, it plays a role in gene transcription and cell division, among other processes (Feng et al. 2010b; Herrera et al. 2017). Therefore, the United Nations considered Zn as a “life-saving commodity,” and this element cannot be stored in the bird’s body (Swain et al. 2016). According to Applegate and Angel (2014) and Council (1994), the Zn requirements in bird diets range from 40 to 75 mg/kg. To meet the Zn requirements, the amount of added Zn should be almost 20- to 30-folds more than that in the normal diets of animals based on the low utilization level of Zn (Bratz et al. 2013). Furthermore, exposure to heat stress conditions increases the need for Zn. Furthermore, a high level of Zn could lead to excess Zn in the fecal matter, causing environmental pollution (Broom et al. 2002). Additionally, it could have a major effect on the balance between the other trace elements in the birds body and reduce the vitamins and nutrients stability (Sahin and Kucuk 2003; Sundaresan et al. 2008; Zhang et al. 2018).

Newly, trace minerals, including Zn nanoparticles, can be successfully used to fulfill the needs of minerals in bird diets (Dosoky et al. 2021; El-Seedi et al. 2019; Mohammed et al. 2020; Sizova et al. 2020) based on their extremely small size and specific physical properties (Biria et al. 2020; Fouda et al. 2021; Radi et al. 2021). Nanoparticles can successfully supply minerals in birds and enhance the growth rate and feed utilization (Abdel-Daim et al. 2019, Abdelsalam et al. 2019, Bhattacharya et al. 2021, Fouda et al. 2020, Gangadoo et al. 2016, Hafez et al. 2017, Ibrahim et al. 2021, Kandeil et al. 2019, Mohammed and Safwat 2020, Oberdörster et al. 2005).

Zinc oxide nanoparticles (ZnO NPs) outweigh conventional Zn sources and positively affect the performance and antioxidant defense of chickens (Ali et al. 2017, El-Katcha et al. 2017b, Eskandani et al. 2021, Mohammadi et al. 2015, Mohammed and Safwat 2013). It could be used as an alternative to antibiotics or as growth promoters (Schmidt 2009). On the other hand, some studies have investigated the toxicity of ZnO NPs in several biological systems, such as bacteria (Sinha et al. 2011) and mammalian cells (Wang et al. 2010). Another study has demonstrated a significantly reduced growth rate of some marine phytoplankton species when ZnO NPs are used in their diet (Miller et al. 2010), explaining the ZnO NPs toxicity in phytoplankton due to the uptake of free Zn ions. In mammalian cells, the ZnO NP toxic effects, like membrane injury, DNA damage, and apoptosis, have been proven (Abdel-Daim et al. 2021; Gojova et al. 2007; Samak et al. 2018). Hence, this study evaluates the efficacy and the possible toxicity of ZnO NPs supplemented in a broiler diet. A feeding trial was taken to investigate their effects on the growth performance, oxidative status, immunologic parameters, and histopathological pictures of internal organs. To the best of the author’s knowledge, this is the first study that evaluates the histopathologic picture of lymphoid organs of ZnO NP-supplemented broiler.

## Materials and methods

This investigation was done at the Faculty of Agriculture (Saba Basha), Poultry Research Laboratory, Alexandria University, Egypt, under the approval of ethical standards of scientific research № *AU: 14/19/12/19/01/06* from Alexandria University, 2019.

### Preparation and characterization of ZnO NPs in powder form

ZnO NPs were produced via the wet chemical technique by utilizing the naturally occurring polysaccharide, namely, sodium alginate, and the precursor, zinc nitrate, Zn(NO_3_)_2_.4H_2_O in the existence of alkaline solution of sodium hydroxide (NaOH) as reported in previous work (Desai et al. 2019; Ishak et al. 2019). At first, 0.5 g of sodium alginate was liquified in 100 ml of dH_2_O comprising 0.2 g of NaOH. The reaction is kept under stirring for about 10 min until complete solubilization. To this end, a solution of 0.1 M of Zn(NO_3_)_2_.4H_2_O was added dropwise, with continuous high-speed stirring for another 30 min. In the end, the formed colloidal solution is left overnight to settle down, and the remarked supernatant solution is carefully separated. The remaining solution was subjected to centrifugation; then the precipitate was collected and washed 3 times with ddH_2_O and C_2_H_6_O to remove the undesired products that may have linked with the formed nanoparticles. The obtained powder is subjected to drying at 80 °C for 24 h, followed by calcination at 600 °C for another hour to warrant the complete conversion of Zn(OH)_2_ to ZnO NPs. For the characterization of ZnO NPs, X-ray diffraction (XRD) was measured under ambient conditions via Siemens D-500 X-ray diffractometer (from 30 mA to 40 kV) bearing a copper (Cu) tube. The morphological description of the ZnO NPs was detected during transmission electron microscopy (TEM) on a JEOL (JEM-1230, Japan); the instrument was with an acceleration voltage of 120 kV.

### Chicken and dietary treatments

A total of 224 1-day-old chicks (male Cobb 500 chicks, white feather chicks) were utilized in this current study. Chicks weighed 42 g on average and were divided into seven groups randomly with 32 birds in each group, which were allotted into 4 replicates (8 birds in each) in a complete randomized design. Chicks were assigned to twelve pens (1.35 × 1.45 m) (Jang et al. 2008). The birds were vaccinated according to Cobb’s company protocol. The starter and growing diets are found in Table 1 according to the National Research Council (Council 1994; Fouda et al. 2021). The treatments were as follows: T1, control, and T2 to T7, the control diet plus the ZnO NPs levels of 5, 10, 20, 40, 60, and 80 ppm/kg diet, respectively. The dose range of ZnO NPs was chosen according to several relevant studies (El-Haliem et al. 2020; Hafez et al. 2020; Ramiah et al. 2019). The dietary experiment started when the chicken was 1-day-old and ended at 35 days of age. The experiment was conducted over a 5-week duration. Feed and water were given ad libitum. The quantity of feed was weighed before being distributed, and the remaining feed in the next week was used to calculate the feed intake of each group. Throughout, the ambient temperature ranged between 28.8 and 33.7 °C, and the relative humidity ranged between 58.0 and 79.01% during the summer of 2019.

**Table 1:** Structure of starter and growing diets (dry matter basis) 1–35-day broilers

Ingredients (%)	Starter (1–28 days)	Grower (29–35 days)
Crushed yellow maize Soy bean meal (48% CP) Sunflower oil Mono Ca(H2PO4)2 Limestone (Ca Co3) NaCl Vit. and mineral mix * DL-Methionine Lysine (C6H14N2O2)	55.750 38.000 2.000 1.600 1.600 0.300 0.300 0.210 0.200	59.590 33.150 3.000 1.600 1.650 0.300 0.300 0.210 0.200
Total	100.00	100.00
Calculated analyses:		
Crude protein (%) ME kcal/kg Crude fat (%) Crude fiber (%) Calcium (Ca %) Phosphorus available ( P %) Methionine (%) Methionine + cysteine (%) Lysine (%)	22.98 3004 2.50 2.71 0.99 0.49 0.57 0.84 1.37	20.98 3104 2.60 2.60 1.00 0.48 0.48 0.83 1.25

^*^Each kilogram of vit. and minerals mixture included: vit. A, 4,000,000I U; vit. D3, 500,000 IU; vit. E, 16.7 g; vit. K, 0.67 g; vit. B1, 0.67 g; vit. B2, 2 g; vit. B6, 0.67 g; vit. B12, 0.004 g; nicotinic acid, 16.7 g; pantothenic acid, 6.67 g; biotin, 0.07 g; folic acid, 1.67 g; choline chloride, 400 g; Zn, 23.3 g; Mn, 10 g; Fe, 25 g; Cu, 1.67 g; I, 0.25 g; Se, 0.033 g; Mg, 133.4 g

### Estimation of growth performance

Chickens were weighed at 1 day of age (the beginning of the test) and weekly for 35 days (the end of the test). Feeding was stopped 12 h before weighing (Li et al. 2007; Marcu et al. 2013). The feed for the day was weighed, and the remaining feed was collected and weighed the next week to calculate the average feed intake (Aydin et al. 2014). The ratio of feed consumption to weight gain was calculated based on the ratio of average feed intake to average gain. Performance index (PI) was determined using the following method: PI = BWG (g) × FER, where BWG is the body weight gain and FER is the feed efficiency ratio (Kalantar et al. 2011). The relative growth rate was estimated according to the following equation (Aggrey 2004):

Growth rate (%) = (*W*_2_ – *W*_1_) × 100/0.5 (*W*_1_ + *W*_2_), where *W*_l_ is the body weight at the beginning of the test and *W*_2_ is the body weight at the last week of the test for which the rate was determined.

### Blood sampling and biochemical index

Eight birds from every treatment (from 4 replicates) were randomly selected and slaughtered. The blood was divided into two parts equally. The first part was stored on ethylenediaminetetraacetic acid (EDTA) to assess the blood hematology (Toghyani et al. 2010), while the second part was centrifuged at 3500 rpm/15 min and used to all biochemical analyses. Serum immunoglobulin fractions (IgM and IgG) were assessed according to Kincade et al. (1970). The phagocytic activity (PA) was calculated according to Hafez et al. (2020). The phagocytic index (PI) was evaluated according to Hafez et al. (2020). Moreover, the hemagglutination inhibition test was utilized to define the humoral antibody titer versus the NDV. Serum lysozyme activity was established through the turbidimetry method described by He et al. (2007). Serum oxidative/antioxidant index was carried out by kits produced by Biodiagnostic, according to Motor et al. (2014).

### Lymphoid organ weight and some carcass traits

Eights chicks from each treatment (4 replicates) were selected, slaughtered to full bleeding, and weighed to calculate the immune organs’ relative weight.

### Nutrient retention

At 5 weeks of age, a digestibility trial was done using 28 males (four cocks per treatment), and each was housed in an individual metabolic cage, which allowed for complete separation and collection of excreta and assessing each dietary treatment. Chemical analyses for nutrients were performed according to the Association of Official Agricultural Chemists (2007). The fecal nitrogen was determined following the procedure outlined in Jacobson et al. (1960) and Jakobsen et al. (2003). Digestibility was determined by accurately measuring feed intake and fecal output. Also, the digestibility coefficient of nutrients was determined.

### Histopathological evaluation

Shortly after slaughter, small specimens from the thymus, spleen, kidneys, liver, and bursa of control and treated chicks were obtained. For fixation, the obtained samples were cleaned and soaked in 10% neutral buffered formalin liquid for 48 h. The paraffin-embedding technique was used to prepare the fixed samples (Saad et al. 2021; Sato et al. 1986, Wright and Manos 1990). Several 4 mm in thick sections were cut and dyed with hematoxylin and eosin on a regular basis. A qualified pathologist (AFK) conducted the blinded assessment and picture capture. A specific digital camera (Leica EC3; Leica, Germany) linked to a microscope was used to take representative micrographs (Leica DM500).

### Ultrastructural evaluations of muscle tissues

Limited specimens of breast muscle were collected immediately after slaughter. Specimens were cut into tiny parts (~ 1 mm^3^) and immediately fixed in 0.1 M phosphate-buffered saline (PBS) for at least 3 h in 3% glutaraldehyde solution C_5_H_8_O_2_ (Merck, Darmstadt, Germany) (pH 7). After two buffer switches, fixed samples were moved to a 1% osmium tetroxide (OsO_4_) solution (Electron Microscope Science, Sigma-Aldrich) for 1 h in 0.1 M PBS (pH = 6.9). After that, the samples were rewashed in 0.1 M PBS for 5 min, dehydrated in increasing ethanol concentrations, and impregnated with Epon embedding resin. The samples were embedded for 48 h at 60 °C and then blocked. For light microscopy, semithin parts were prepared and stained with 1% simple toluidine blue. After that, the ultrathin parts (50–80 nm) were cut from the chosen areas and put on copper grids (200 mesh). Finally, segment comparing was carried out with uranyl acetate dihydrate (2%) and lead citrate. Tissues were investigated and photographed using a JEM-1220 transmission electron microscope (TEM; JEOL, Tokyo, Japan).

### Gene expression

A real-time polymerase chain reaction was used to evaluate the precise expression of inflammatory genes (*IL-B1* and *TNF-α*) in muscle tissues (Dosoky et al. 2021; Saad et al. 2021). TRIzol reagent (Invitrogen/Life Technologies, Carlsbad, CA, USA) and NanoDrop for quantification were utilized for RNA extraction from ~ 100 mg muscle. A260 or A260/A280 RNA samples were used to synthesize DNA with a cDNA synthesis package (Fermentas, Waltham, MA, USA). Table 2 shows the primers and housekeeping gene.

**Table 2:** Primer sequences for the genes

	Forward	Reverse	Accession number
Bactin	GTCCACCTTCCAGCAGATGT	ATAAAGCCATGCCAATCTCG	396,526
ILB1	AGGTGAGAGTCCCGAGTCC	GTAGGTGGCGATGTTGACCT	AJ245728
TNFα	CAGGACAGCCTATGCCAACA	AACTCATCTGAACTGGGCGG	HQ739087

#### Statistical analysis

To analyze the effects of ZnO NP levels on the dependent variables, SPSS version 16 (SPSS, Inc., Chicago, IL, USA) was used based on one-way ANOVA. Data are represented as means. Significant variation intergroup means were obtained (Duncan 1955). Statistical significance was accepted at *P* ≤ *0.05*.

## Results

### Characterization ZnO NPs

**Fig. 1:**
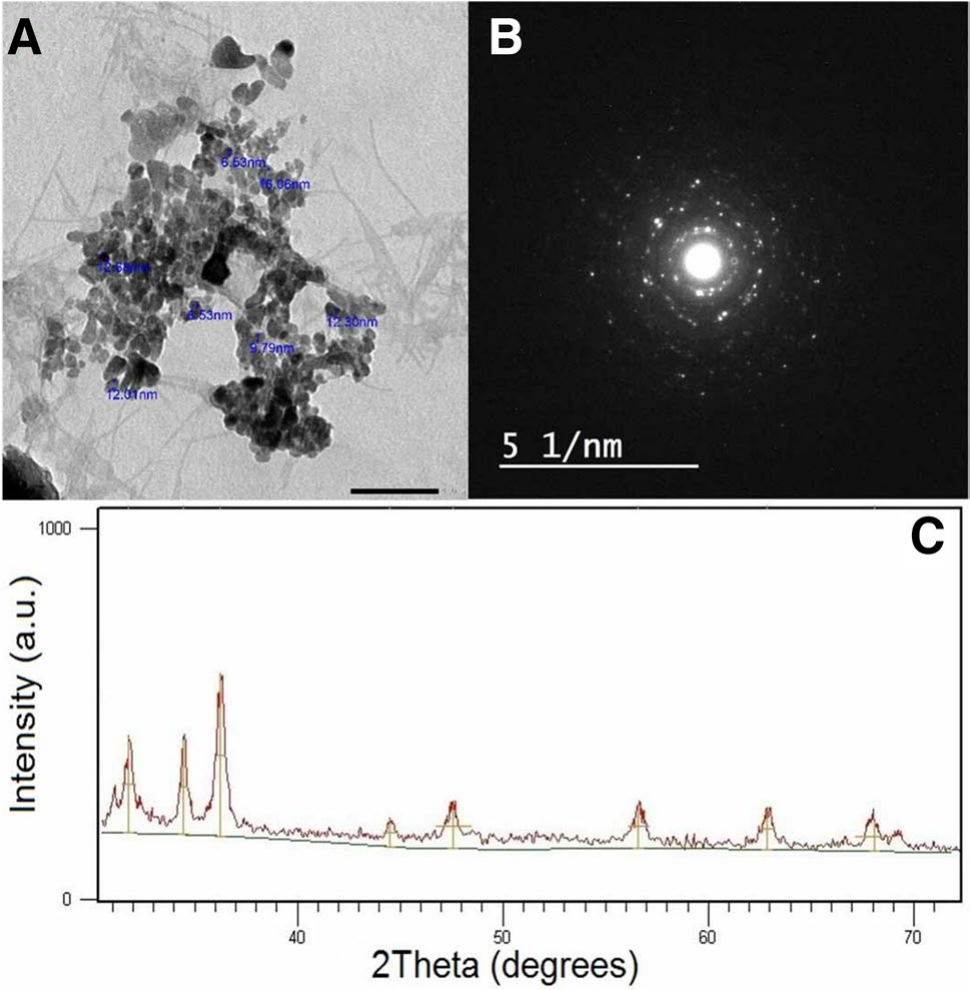
Characterization of ZnO NPs showing **A** TEM, **B** selected area electron diffraction (SAED), and **C** X-ray diffraction (XRD)

The morphology, particle size structure, and distribution of ZnO NPs were evaluated using TEM. The instrument was adjusted within an acceleration voltage of 120 kV. The findings are outlined in Fig. 1A. As it is evident, the formed particles are nearly spherical with a comparatively narrow size distribution, as observed in Fig. 1A. The major sizes were in the range of 6.53 nm. In addition, there was a plausible portion of ~ 26% with the total sizes of ~ 12.3 nm, which might be attributed to the tendency of the formed particles to aggregate to larger sizes. The polycrystalline nature of ZnO NPs is detected by the resultant selected area electron diffraction (Fig. 1B). Once electron diffraction is conceded on a limited number of crystals, the exterior of the discrete points in the circle pattern affirmed that most ZnO NPs are more or less single-crystalline materials and are predominately along with ZnO NP direction, as ordinarily originated for the ZnO crystal lattice. The X-ray diffraction (XRD) study of the generated ZnO-based NPs is depicted in Fig. 1C. The broad XRD signals designate that the generated ZnO-based powder is composed of nanoscale particles. The diffraction XRD signals at 31.79°, 34.40°, 36.21°, 44.51°, 47.68°, 56.75°, 62.95°, 68.15°, and 69.07° have been reported previously and indexed as a hexagonal wurtzite structure of ZnO-based NPs with lattice constants *a* = *b* = 0.325 nm and *c* = 0.519 nm. The XRD spectra proved that the produced powder of ZnO-based NPs is pure because no characteristic XRD signals were detected other than the ZnO signals.

### Growth performance parameters

The effects of ZnO NP supplementation on the growth performance of broilers are shown in Table 3. Results indicated that the average live BW of broilers did not differ significantly (*P* > *0.05*) between treatment groups. Treatment with the 5 ppm ZnO NPs/kg diet recorded the highest LBW at 21 and 35 days. At 35 days, the BW was 1940.90 g at the 5 ppm/kg level and improved by 21.60 g compared to the control (Table 3). BWG increased by 873.02 g for the age of 1 to 21 days under the 5 ppm/kg level and by 1898 g for the period of 21 to 35 days. Results revealed no significant impacts for FI and FCR among the treated and control birds. Furthermore, the FCR had the lowest nonsignificant values (*P* > *0.05*) compared to the control birds, for example, 1.410 g in the control birds and under a level of 5 ppm/kg for 1 to 35 days. In the same trend, growth rate (%) and PI were found (Table 3).

**Table 3:** Effect of zinc nanoparticles on the productive performance of boiler chickens from 1 to 5 weeks of age

Items	Treatments*							*P* value
	T1	T2	T3	T4	T5	T6	T7	
Live body weight (g/bird/period)								
1 day	42.73 ± 0.65	42.65 ± 0.51	42.95 ± 0.58	42.61 ± 1.18	42.04 ± 0.53	42.8 ± 0.46	42.69 ± 0.48	0.949
21 days	881.66 ± 11.65	915.66 ± 20.33	882.62 ± 13.15	893.00 ± 21.37	896.57 ± 14.17	902.17 ± 15.60	892.34 ± 12.83	0.552
35 days	1919.30 ± 34.46	1940.90 ± 31.24	1891.30 ± 23.87	1906.30 ± 48.57	1877.00 ± 32.27	1927.30 ± 31.84	1846.60 ± 27.71	0.757
Body weight gain (g/bird/period)								
1–21 days	838.94 ± 2.15	873.02 ± 11.92	839.42 ± 4.82	850.39 ± 14.06	851.16 ± 13.35	853.70 ± 11.49	849.65 ± 5.44	0.240
21–35 days	1037.60 ± 33.95	1025.20 ± 10.51	1008.90 ± 15.81	1013.30 ± 64.69	983.67 ± 20.09	1030.70 ± 27.22	954.25 ± 26.55	0.621
1–35 days	1876.54 ± 35.26	1898.22 ± 20.17	1848.32 ± 16.26	1863.69 ± 54.81	1834.83 ± 30.51	1884.40 ± 17.31	1803.90 ± 31.54	0.478
Feed intake (g/bird/ period)								
1–21 days	1107.10 ± 13.86	1151.10 ± 18.96	1104.70 ± 17.37	1123.20 ± 10.85	1123.10 ± 13.69	1123.20 ± 11.79	1086.90 ± 5.81	0.091
21–35 days	1533.10 ± 10.69	1522.40 ± 5.08	1538.90 ± 6.07	1553.70 ± 8.18	1517.20 ± 1.64	1567.00 ± 46.10	1513.40 ± 16.00	0.285
1–35 days	2640.20 ± 8.67	2673.50 ± 14.63	2643.60 ± 22.33	2676.90 ± 18.95	2640.30 ± 11.98	2690.20 ± 35.01	2600.30 ± 21.60	0.070
Feed conversion ratio								
1–21 days	1.320 ± 0.01	1.318 ± 0.02	1.318 ± 0.01	1.323 ± 0.02	1.323 ± 0.01	1.317 ± 0.00	1.278 ± 0.00	0.568
21–35 days	1.485 ± 0.06	1.488 ± 0.01	1.528 ± 0.01	1.555 ± 0.08	1.543 ± 0.03	1.520 ± 0.02	1.590 ± 0.03	0.703
1–35 days	1.410 ± 0.02	1.410 ± 0.01	1.430 ± 0.00	1.440 ± 0.03	1.440 ± 0.02	1.427 ± 0.01	1.440 ± 0.01	0.891
Growth rate (%)								
1–21 days	161.08 ± 0.38	161.86 ± 0.38	161.72 ± 0.02	161.1 ± 0.67	162.38 ± 0.71	161.28 ± 1.88	161.39 ± 0.60	0.881
21–35 days	61.71 ± 1.06	59.97 ± 0.34	60.66 ± 0.45	60.61 ± 2.54	59.69 ± 0.47	61.14 ± 1.39	58.07 ± 1.08	0.556
1–35 days	180.48 ± 0.46	180.45 ± 0.16	180.6 ± 0.14	180.12 ± 0.61	180.88 ± 0.30	180.81 ± 0.96	179.74 ± 0.31	0.636
Performance index								
1–35 days	136.52 ± 5.06	137.86 ± 3.24	132.24 ± 1.20	132.86 ± 7.22	130.48 ± 3.73	135.02 ± 1.71	128.12 ± 3.47	0.693

^*^T1, control; *T2* (control + 5 ppm ZnO NPs/kg diet), *T3* (control + 10 ppm ZnO NPs/kg diet), *T4* (control + 20 ppm ZnO NPs/kg diet), *T5* (control + 40 ppm ZnO NPs/kg diet), *T6* (control + 60 ppm ZnO NPs/kg diet), *T7* (control + 80 ppm ZnO NPs/kg diet)

### Biochemical and hematological parameters

Among hematological and immunological variables, WBC and RBC counts, PCV, lymphocytes (%), heterophils (%), lymphocytes/heterophils ratio, and monocytes were significantly (*P* ≤ *0.05*) affected by ZnO NP supplementation. The group given the 10 ppm ZnO NPs/kg diet had the highest RBC counts compared to the other treated groups. However, Hb concentration, basophils (%), and eosinophils (%) were not significantly influenced by different treatments (Table 4).

**Table 4:** Effect of zinc nanoparticles on some hematological and immunological index of chickens at 5 weeks of age

Items	Treatments*							*P* value
	T1	T2	T3	T4	T5	T6	T7	
Red blood cells (RBCs10^6^/mm^3^)	1.57^a^ ± 0.02	1.53^a^ ± 0.05	1.60^a^ ± 0.08	1.57^a^ ± 0.06	1.27^b^ ± 0.06	1.20^b^ ± 0.04	1.17^b^ ± 0.02	0.001
White blood cells (WBCs10^3^/m^3^)	21.00^b^ ± 0.40	21.00^b^ ± 0.05	21.00^b^ ± 0.51	21.33^b^ ± 0.62	21.67^b^ ± 0.62	24.33^a^ ± 0.47	23.67^a^ ± 0.62	0.001
Hemoglobin (Hb g/dl)	10.67 ± 0.24	10.33 ± 0.47	11.00 ± 0.41	10.67 ± 0.24	11.67 ± 0.24	10.33 ± 0.47	10.67 ± 0.24	0.152
Packed cell volume (PCV %)	33.67^ab^ ± 0.24	32.67^b^ ± 0.24	33.67^ab^ ± 0.62	33.33^ab^ ± 0.24	35.00^a^ ± 0.41	31.67^b^ ± 1.31	33.67^ab^ ± 0.62	0.048
Lymphocytes%	62.33^bc^ ± 0.62	64.67^a^ ± 0.62	62.33^bc^ ± 0.62	64.00^ab^ ± 0.41	63.33^abc^ ± 0.62	61.33^c^ ± 0.94	59.00^d^ ± 0.41	0.001
Heterophils%	32.70^b^ ± 1.24	30.73^b^ ± 0.61	32.10^b^ ± 0.82	30.00^b^ ± 0.82	30.00^b^ ± 1.08	32.03^b^ ± 1.61	36.00^a^ ± 0.82	0.008
H/L ratio	0.525^b^ ± 0.02	0.476^b^ ± 0.01	0.516^b^ ± 0.02	0.469^b^ ± 0.02	0.474^b^ ± 0.02	0.524^b^ ± 0.03	0.611^a^ ± 0.02	0.001
Monocytes%	3.00^b^ ± 0.71	3.00^b^ ± 0.04	3.67^ab^ ± 0.24	3.67^ab^ ± 0.62	5.67^a^ ± 0.62	5.00^ab^ ± 1.08	3.00^b^ ± 0.41	0.036
Basophils%	0.67 ± 024	0.67 ± 0.24	1.00 ± 0.00	1.00 ± 0.00	0.333 ± 0.24	0.668 ± 0.24	1.00 ± 0.00	0.111
Eosinophils%	1.30 ± 0.25	0.933 ± 0.02	0.900 ± 0.00	1.33 ± 0.24	0.668 ± 0.47	0.968 ± 0.02	1.00 ± 0.00	0.387
Humoral immunity parameters								
Phagocytic activity (PA)	19.33^b^ ± 0.62	18.67^b^ ± 0.24	18.67^b^ ± 0.24	21.00^a^ ± 0.41	21.00^a^ ± 0.41	21.00^a^ ± 0.41	19.33^b^ ± 0.62	0.001
Phagocytic activity (PA)	1.97^a^ ± 0.06	1.77^c^ ± 0.02	1.83^bc^ ± 0.02	2.03^a^ ± 0.02	1.93^ab^ ± 0.02	1.97^a^ ± 0.06	1.77^c^ ± 0.02	0.001
Antibody titter against NDV; HI	6.33 ± 0.47	6.00 ± 0.00	7.00 ± 0.00	6.33 ± 0.47	6.33 ± 0.24	6.00 ± 0.41	5.67 ± 0.24	0.173
Immunoglobulin M (mg/dl)	23.53^a^ ± 0.09	23.33^ab^ ± 0.01	22.93^ cd^ ± 0.09	23.53^a^ ± 0.17	23.27^abc^ ± 0.12	23.13^bcd^ ± 0.06	22.90^d^ ± 0.08	0.002
Immunoglobulin G (mg/dl)	973.67^a^ ± 1.03	972.00^ab^ ± 1.47	972.00^ab^ ± 0.71	972.33^ab^ ± 1.31	972.33^ab^ ± 1.31	967.33^bc^ ± 1.93	966.33^c^ ± 2.80	0.028
Lysosome U/L	4.117 ± 0.01	4.117 ± 0.01	4.123 ± 0.01	4.113 ± 0.00	4.107 ± 0.01	4.113 ± 0.01	4.107 ± 0.01	0.116
Interleukin 6 ug/mL	40.67 ± 0.24	40.07 ± 0.05	40.33 ± 0.24	40.33 ± 0.24	40.67 ± 0.24	40.00 ± 0.41	40.33 ± 0.24	0.419

^*^T1, control; *T*_*2*_ (control + 5 ppm ZnO NPs/kg diet), *T3* (control + 10 ppm ZnO NPs/kg diet), *T4* (control + 20 ppm ZnO NPs/kg diet), *T5* (control + 40 ppm ZnO NPs/kg diet), *T6* (control + 60 ppm ZnO NPs/kg diet), *T7* (control + 80 ppm ZnO NPs/kg diet)

^a–^^d^Means in the same row having several letters are significantly different. (*P* ≤ *0.05*)

### Immunological parameters

Data in Table 4 showed that significant differences (*P* ≤ *0.05*) between groups were obtained for PA, PI, IgM, and IgG, where the best value was recorded in the groups given 5 and 10 ppm ZnO NPs/kg diet, followed by the group given 20 ppm ZnO NPs/kg diet. However, antibody titers against the NDA, lysosomes, and interleukin (IL)-6 were not significantly affected by different treatments.

### Biochemical parameters

Data obtained on serum biochemical estimates in chicks as affected by ZnO NPs are shown in Table 5. Results showed that serum albumin, total protein, globulin, and albumin/globulin ratio were not significantly different in all groups. Results also showed that serum ALP was insignificantly (*P* ≤ *0.05*) enhanced in all treated groups, except the group given the 20 ppm ZnO NPs/kg diet. In contrast, serum AST was significantly (*P* ≤ *0.05*) increased in the groups given the 40 and 80 mg ZnO NPs/kg diet compared to the control.

**Table 5:** Effect of zinc nanoparticles on some blood serum constituents of boiler chickens at 5 weeks of age

Items	Treatments*							*P* value
	T1	T2	T3	T4	T5	T6	T7	
Total protein (g/dl)	5.77 ± 0.23	5.84 ± 0.10	5.93 ± 0.12	6.13 ± 0.02	5.90 ± 0.04	6.10 ± 0.04	5.70 ± 0.08	0.108
Albumin (g/dl)	3.03 ± 0.09	3.07 ± 0.06	3.13 ± 0.06	3.10 ± 0.04	3.17 ± 0.04	3.10 ± 0.04	3.03 ± 0.02	0.431
Globulin (g/dl)	2.73 ± 0.09	2.77 ± 0.15	2.80 ± 0.07	3.03 ± 0.00	2.73 ± 0.08	3.00 ± 0.07	2.67 ± 0.06	0.112
A/G	1.11 ± 0.00	1.11 ± 0.01	1.12 ± 0.00	1.02 ± 0.00	1.16 ± 0.11	1.03 ± 0.01	1.14 ± 0.00	0.202
Alkaline phosphatase (IU/L)	1113.30 ± 2.01	1113.00 ± 2.12	1113.00 ± 2.12	1111.00 ± 0.00	1116.00 ± 2.12	1113.30 ± 2.01	1116.00 ± 2.12	0.544
Alanine aminotransferase (U/L)	64.00^a^ ± 0.82	61.33^b^ ± 0.62	64.33^a^ ± 0.85	62.33^ab^ ± 0.62	61.00^b^ ± 0.40	63.33^ab^ ± 0.94	62.00^ab^ ± 1.08	0.043
Aspartate aminotransferase (U/L)	54.67^ cd^ ± 0.47	53.33^d^ ± 1.02	55.33^bcd^ ± 0.47	55.00b^cd^ ± 1.47	60.67a ± 0.23	56.67^bc^ ± 0.94	58.00^ab^ ± 1.47	0.043
Total lipids (mg/dl)	569.87 ± 0.01	571.53 ± 2.02	578.07 ± 15.20	576.53 ± 2.30	577.40 ± 2.30	574.87 ± 1.02	585.93 ± 1.07	0.294
Total cholesterol (mg/l)	212.67 ± 4.13	211.00 ± 1.54	212.67 ± 1.54	210.00 ± 2.16	210.67 ± 0.85	210.33 ± 0.23	212.33 ± 2.09	0.923
Low-density lipoprotein (mg/l)	40.67^ab^ ± 0.18	36.00^c^ ± 1.41	37.00^bc^ ± 1.41	40.33^ab^ ± 0.23	39.00^abc^ ± 1.41	38.33^abc^ ± 0.85	42.00^a^ ± 1.79	0.019
High-density lipoprotein (mg/l)	101.33^a^ ± 0.95	101.33^a^ ± 0.70	102.00^a^ ± 0.70	99.00^b^ ± 0.40	97.33^bc^ ± 0.62	103.00^a^ ± 1.22	96.00^e^ ± 0.00	0.001
Triglycerides(mg/dl)	182.67^c^ ± 2.49	186.00^bc^ ± 1.24	188.67^ab^ ± 1.24	189.33^ab^ ± 2.24	192.00^a^ ± 1.47	186.00^bc^ ± 0.81	193.00^a^ ± 1.08	0.002
Uric acid (mg/dl)	3.73 ± 0.19	03.50 ± 0.23	03.67 ± 0.23	03.73 ± 0.18	03.83 ± 0.23	03.67 ± 0.23	4.33 ± 0.23	0.241
Creatinine (mg/dl)	1.07^bc^ ± 0.02	1.00^c^ ± 0.02	1.13^abc^ ± 0.02	1.17^ab^ ± 0.04	1.23^a^ ± 0.06	1.13^abc^ ± 0.02	1.23^a^ ± 0.06	0.011
Catalase (mu/ ml)	410.33^ab^ ± 10.22	410.00ab ± 11.20	400.00^b^ ± 10.01	410.67^a^ ± 9.01	350.33^c^ ± 0.55	400.67^ab^ ± 1.00	360.00^c^ ± 0.25	0.001
Malondialdehyde (nmol/ ml)	10.33^c^ ± 0.24	10.43^c^ ± 0.30	10.67^bc^ ± 0.23	11.33^abc^ ± 0.62	11.67^ab^ ± 0.23	11.67^ab^ ± 0.47	12.10^a^ ± 0.07	0.010
Total antioxidant capacity (mg/dl)	0.413^a^ ± 0.01	0.413^a^ ± 0.00	0.411^ab^ ± 0.00	0.410^abc^ ± 0.01	0.410^abc^ ± 0.21	0.408^bc^ ± 0.00	0.407^c^ ± 0.01	0.020
Zn (mg/dl)	72.00^d^ ± 0.71	73.33^d^ ± 1.02	77.67^c^ ± 1.24	79.33^bc^ ± 0.47	82.00^ab^ ± 1.47	83.67^a^ ± 1.31	80.00^abc^ ± 1.87	0.000
Ca (mg/dl)	9.57^a^ ± 0.01	9.43^ab^ ± 1.01	9.67^a^ ± 0.64	9.40^abc^ ± 0.05	9.53^ab^ ± 0.12	9.07^c^ ± 1.01	9.20^bc^ ± 0.00	0.012
P (mg/dl)	4.47^a^ ± 0.00	4.03^d^ ± 0.00	4.10^bcd^ ± 0.01	4.07^ cd^ ± 0.24	4.30^ab^ ± 0.32	4.27^abc^ ± 0.00	4.37^a^ ± 0.01	0.001

^*^T1, control; *T2* (control + 5 ppm ZnO NPs/kg diet), *T3* (control + 10 ppm ZnO NPs/kg diet), *T4* (control + 20 ppm ZnO NPs/kg diet), *T5* (control + 40 ppm ZnO NPs/kg diet), *T6* (control + 60 ppm ZnO NPs/kg diet), *T7* (control + 80 ppm ZnO NPs/kg diet)

^a–^^d^Means in the same row having several letters are significantly different (*P* ≤ *0.05*)

ALT was significantly (*P* ≤ *0.05*) decreased by different treatments, and the lowest value was obtained in the group given the 40 ppm ZnO NPs/kg diet. Serum total lipid and cholesterol concentrations were insignificantly (*P* ≤ *0.05*) decreased due to the addition of different levels of ZnO NPs compared with control. However, triglyceride and HDL concentrations were significantly (*P* ≤ *0.05*) decreased. In addition, LDL levels were significantly (*P* ≤ *0.05*) improved compared to the control. Serum uric acid showed a nonsignificant difference between the control and treated groups. However, creatinine was significantly (*P* ≤ *0.05*) increased in groups given the 40 and 80 ppm ZnO NPs/kg diet. The lowest creatinine and uric acid concentrations were recorded in groups given the 5 and 10 ppm ZnO NPs/kg diet, respectively.

### Oxidative parameters

Serum CAT was significantly (*P* ≤ *0.05*) decreased in groups the given 40 and 80 ppm ZnO NPs/kg diet compared to the group control. TAC showed a significant reduction (P ≤ 0.01) in broilers given the 60 and 80 ppm ZnO NPs/kg diet. Additionally, serum lipid peroxide (MDA) concentrations were significantly (*P* ≤ *0.01*) increased in groups given the 40, 60, and 80 ppm ZnO NPs/kg diet (Table 5).

### Organ weight and digestibility

Table 5 shows a significant (*P* ≤ *0.05*) increase in serum Zn, calcium, and inorganic phosphorous concentrations observed with increased ZnO NP levels in the diet. The carcass relative weights, proventriculus gland, liver, gizzard, spleen, pancreas, intestine, cecum, bursa, and thymus were not significantly (*P* ≤ *0.05*) affected by ZnO NP supplementation. However, the relative weights of the gizzard, spleen, pancreas, and thymus were significantly (*P* ≤ *0.001* or *0.01*) affected by ZnO NP treatments (Table 6). Data regarding the effects of including different ZnO NP levels in broiler diets on the digestibility coefficients of nutrients are shown in Table 7. Different supplementations had nonsignificant effects on the digestibility of nutrients (*P* ≤ *0.05*).

**Table 6:** Effect of zinc nanoparticles on carcass traits of chickens at 35 days

Items	Treatments*							*P* value
	T1	T2	T3	T4	T5	T6	T7	
Carcass weight %	75.54 ± 1.03	76.48 ± 0.67	74.35 ± 0.82	76.75 ± 1.05	76.72 ± 1.22	75.14 ± 1.02	73.32 ± 1.78	0.290
Proventriculus gland %	0.339 ± 0.02	0.304 ± 0.00	0.313 ± 0.02	0.314 ± 0.03	0.236 ± 0.01	0.328 ± 0.02	0.288 ± 0.02	0.121
Liver weight %	1.59 ± 0.08	1.57 ± 0.17	1.41 ± 0.04	1.56 ± 0.09	1.56 ± 0.06	1.33 ± 0.06	1.40 ± 0.10	0.344
Gizzard %	2.16^a^ ± 0.15	1.96^ab^ ± 0.05	2.19^a^ ± 0.12	1.94^ab^ ± 0.18	2.11^a^ ± 0.15	2.09^a^ ± 0.10	1.61^b^ ± 0.05	0.027
Spleen %	0.144^a^ ± 0.01	0.120^ab^ ± 0.00	0.098^bc^ ± 0.01	0.098^bc^ ± 0.01	0.086^bc^ ± 0.00	0.089^bc^ ± 0.00	0.070^c^ ± 0.01	0.005
Heart %	0.369 ± 0.01	0.470 ± 0.03	0.434 ± 0.02	0.361 ± 0.01	0.374 ± 0.02	0.414 ± 0.03	0.399 ± 0.02	0.088
Pancreas %	0.154^ cd^ ± 0.00	0.185^abc^ ± 0.01	0.149^d^ ± 0.00	0.171^bcd^ ± 0.01	0.185^abc^ ± 0.00	0.198^ab^ ± 0.00	0.206^a^ ± 0.01	0.008
Intestines %	2.87 ± 0.07	2.41 ± 0.06	2.39 ± 0.13	2.05 ± 0.31	2.50 ± 0.32	2.41 ± 0.17	2.36 ± 0.08	0.215
Cecum %	0.651 ± 0.05	0.591 ± 0.06	0.548 ± 0.04	0.648 ± 0.06	0.458 ± 0.03	0.545 ± 0.06	0.524 ± 0.06	0.223
Bursa %	0.119 ± 0.02	0.141 ± 0.01	0.110 ± 0.02	0.093 ± 0.01	0.066 ± 0.00	0.102 ± 0.01	0.088 ± 0.01	0.175
Thymus %	0.400^a^ ± 0.02	0.446^a^ ± 0.04	0.260^bc^ ± 0.01	0.373^ab^ ± 0.06	0.187^c^ ± 0.07	0.244^bc^ ± 0.02	0.222^c^ ± 0.02	0.003

^*^T1, control; *T2* (control + 5 ppm ZnO NPs/kg diet), *T3* (control + 10 ppm ZnO NPs/kg diet), *T4* (control + 20 ppm ZnO NPs/kg diet), *T5* (control + 40 ppm ZnO NPs/kg diet), *T6* (control + 60 ppm ZnO NPs/kg diet), *T7* (control + 80 ppm ZnO NPs/kg diet)

^a–^^d^Means in the same row having several letters are significantly different (*P* ≤ *0.05*)

**Table 7:** Effect of zinc nanoparticles (ZnO NPs) on digestibility percentage of nutrients of boiler chickens at 5 weeks of age

Items	Treatments*							*P* value
	T1	T2	T3	T4	T5	T6	T7	
Dry matter	74.12 ± 0.82	75.22 ± 1.10	74.45 ± 2.15	75.34 ± 2.17	75.92 ± 2.22	74.41 ± 2.29	73.17 ± 3.07	0.421
Organic matter	85.43 ± 1.21	85.61 ± 1.41	85.71 ± 1.57	85.58 ± 1.41	85.92 ± 1.02	85.57 ± 0.79	85.56 ± 1.47	0.201
Crude protein	93.56 ± 0.98	94.08 ± 0.38	93.24 ± 0.84	93.71 ± 0.80	93.13 ± 1.48	93.71 ± 0.85	92.44 ± 1.08	0.274
Ether extract	82.02 ± 0.95	82.28 ± 1.04	82.42 ± 0.54	81.33 ± 0.98	81.34 ± 1.09	81.35 ± 0.52	81.83 ± 0.65	0.258
Crude fiber	21.89 ± 0.81	21.49 ± 0.61	21.42 ± 0.26	21.56 ± 0.97	21.72 ± 1.30	21.21 ± 0.74	21.33 ± 1.29	0.411
Nitrogen free extract	85.58 ± 1.74	85.15 ± 1.52	86.10 ± 1.41	85.92 ± 1.61	84.91 ± 2.34	84.90 ± 2.14	85.16 ± 1.67	0.314

^*^T1, control; *T2* (control + 5 ppm ZnO NPs/kg diet), *T3* (control + 10 ppm ZnO NPs/kg diet), *T4* (control + 20 ppm ZnO NPs/kg diet), *T5* (control + 40 ppm ZnO NPs/kg diet), *T6* (control + 60 ppm ZnO NPs/kg diet), *T7* (control + 80 ppm ZnO NPs/kg diet)

### Histopathological evaluations

#### Liver

**Fig. 2:**
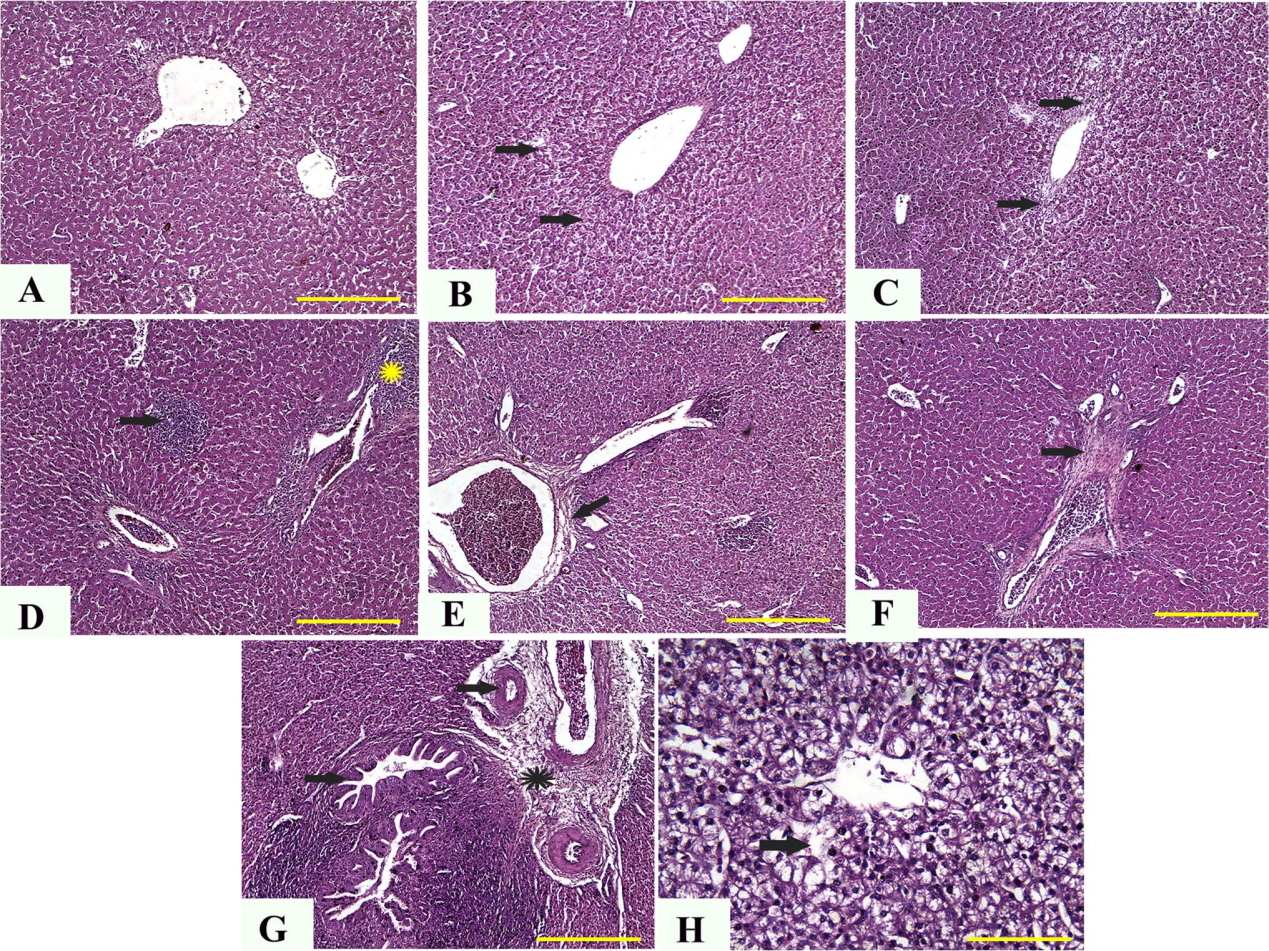
Representative photomicrographs from the liver of chicken treated with several levels of ZnO NPs for 5 weeks: H&E staining. The control group (**A**), broilers treated with 5 ppm of ZnO NPs (**B**), broilers treated with 10 ppm of ZnO NPs (**C**), broilers treated with 20 ppm of ZnO NPs (**D**), broilers treated with 40 ppm of ZnO NPs (**E**), broilers treated with 60 ppm of ZnO NPs (**F**), and broilers treated with 80 ppm of ZnO NPs (**G**, **H**) showing normal hepatic tissue (**A**), infrequent mild centrilobular hydropic vacuolization (arrows) (**B**, **C**), multifocal aggregation of mononuclear cells (arrow) and thickening of portal areas (yellow star) (**D**), fibroplasia (arrow) (**E**, **F**), pronounced thickening of the periportal fibrous tissues (star), newly developed bile ductules, and papillary hyperplasia (arrows) (**G**), and diffuse vacuolization of hepatic lobules (arrow) (**H**). ZnO NP, zinc oxide nanoparticle; H&E, hematoxylin and eosin. Scale bars = 100 μm (× 10) for photomicrographs (**A**–**G**) and 50 μm (× 40) for photomicrographs (**H**)

The histopathological analysis of control chickens showed normal hepatic tissue with the natural structure of hepatic lobules, portal areas, and central veins and no specific lesions (Fig. 2A). In the meantime, chickens given 5 mg (Fig. 2B) and 10 mg (Fig. 2C) ZnO NPs had infrequent moderate centrilobular hydropic vacuolization. The pathological analysis of liver tissues from chickens given 20, 40, and 60 mg ZnO NPs revealed multifocal aggregation of mononuclear cells (Fig. 2D), portal obstruction and thickening of portal areas with infiltrated mononuclear cells (Fig. 2E), and fibroplasia (Fig. 2F). Furthermore, in chicks given 80 mg ZnO NPs, there was pronounced thickening of periportal fibrous tissues with newly developed bile ductile, multifocal areas of coagulative necrosis, and diffuse vacuolization of hepatic lobules (Fig. 2G and H).

#### Kidney

**Fig. 3:**
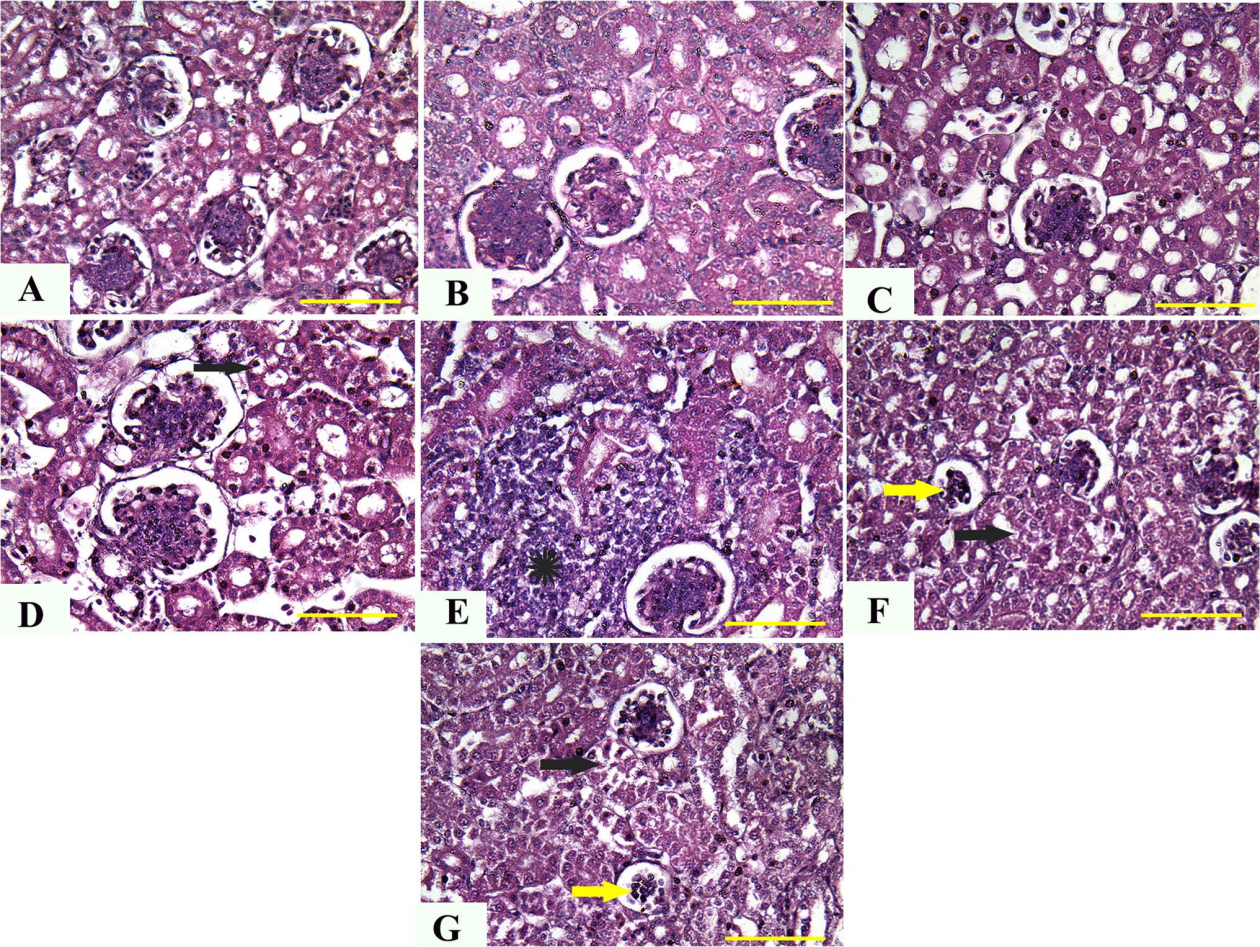
Representative photomicrographs from the kidney of chicken treated with several levels of ZnO NPs for 5 weeks: H&E staining. The control group (**A**), broilers treated with 5 ppm of ZnO NPs (**B**), broilers treated with 10 ppm of ZnO NPs (**C**), broilers treated with 20 ppm of ZnO NPs (**D**), broilers treated with 40 ppm of ZnO NPs (**E**), broilers treated with 60 ppm of ZnO NPs (**F**), and broilers treated with 80 ppm of ZnO NPs (**G**) showing normal histologic structures of renal tissue (**A**–**C**), moderate vacuolization and degeneration of the renal epithelium (arrow) (**D**), significant infiltration of interstitial mononuclear inflammatory cells (star) (**E**), and marked vacuolization and degeneration of renal epithelium (black arrow) together with atrophied glomerulus (yellow arrow) (**G**). ZnO NP, zinc oxide nanoparticle; H&E, hematoxylin and eosin. Scale bars = 50 μm (× 40)

Histopathological analysis of kidney tissues indicated that the kidneys of control chickens and those given 5 and 10 mg ZnO NPs had nearly average histological structures of the glomerulus, renal epithelium, and renal tubules (Fig. 3A–C). In contrast, renal tissues from chicks given 20 mg ZnO NPs demonstrated moderate vacuolization and degeneration of the renal epithelium (Fig. 3D). Furthermore, kidneys from chickens supplemented with 40, 60, and 80 mg ZnO NPs demonstrated significant infiltration of interstitial mononuclear inflammatory cells (Fig. 3E) and significant vacuolization and degeneration of the renal epithelium (Fig. 3F and G).

#### Bursa of Fabricius

**Fig. 4:**
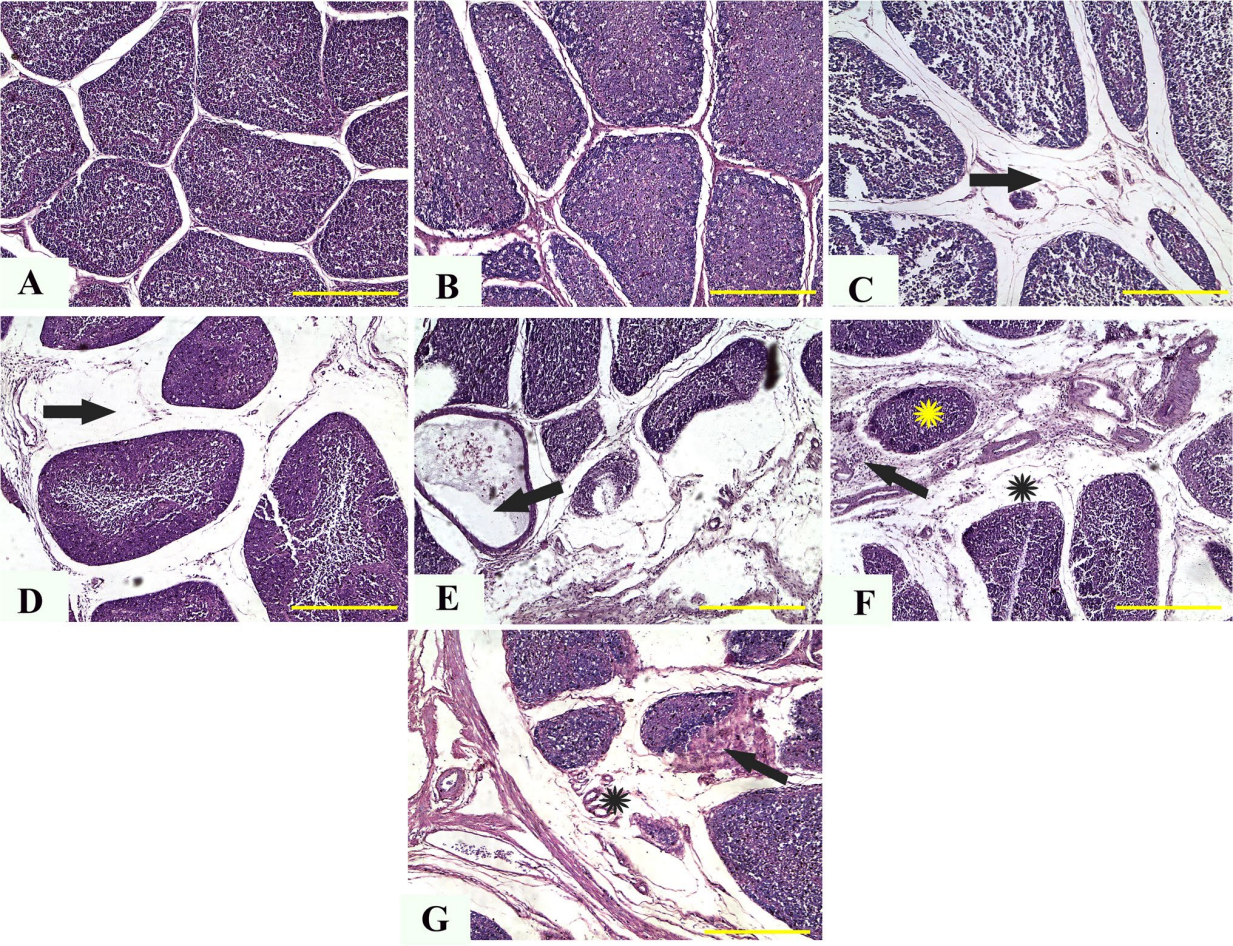
Representative photomicrographs from the bursa of broilers treated with different concentrations of ZnO NPs for 35 days: H&E staining. The control group (**A**), broilers treated with 5 ppm of ZnO NPs (**B**), broilers treated with 10 ppm of ZnO NPs (**C**), broilers treated with 20 ppm of ZnO NPs (**D**), broilers treated with 40 ppm of ZnO NPs (**E**), broilers treated with 60 ppm of ZnO NPs (**F**), and broilers treated with 80 ppm of ZnO NPs (**G**) showing normal histological archicture of bursa (**A**, **B**), reduced number and size of follicles, reduced medullary cell populations, and widened interfollicular space with edema (arrows) (**C**, **D**), atrophy of the majority of the bursa follicles, with the atrophied follicles comprising single cystic structure containing tissue debris (arrow) (**E**), atrophied follicle (yellow star), interfollicular edema (black star), inflammatory filtrates (arrow) (**F**), interfollicular edema (black star), and focal coagulative necrosis of follicle (arrow) ZnO NPs, zinc nanoparticle; H&E, hematoxylin and eosin. Scale bars = 100 μm (× 10)

Control and treated chickens (5 mg) showed normal histological architecture of bursa in normal size and follicles number, normal intensity of medullary and cortical lymphocytic populations, and distinct corticomedullary junction (Fig. 4A and B). However, chickens given 10 or 20 mg ZnO NPs showed reduced number and size of follicles, reduced medullary cell populations, and widened interfollicular space with edema (Fig. 4C and D). However, tissue analysis of chickens given 40, 60, and 80 mg ZnO NPs showed atrophy of most bursa follicles, with atrophic follicles comprising a single cystic structure containing tissue debris (Fig. 4E). Furthermore, interfollicular edema and inflammatory filtrates were abundant in almost all parts (Fig. 4F and G).

#### Spleen

**Fig. 5:**
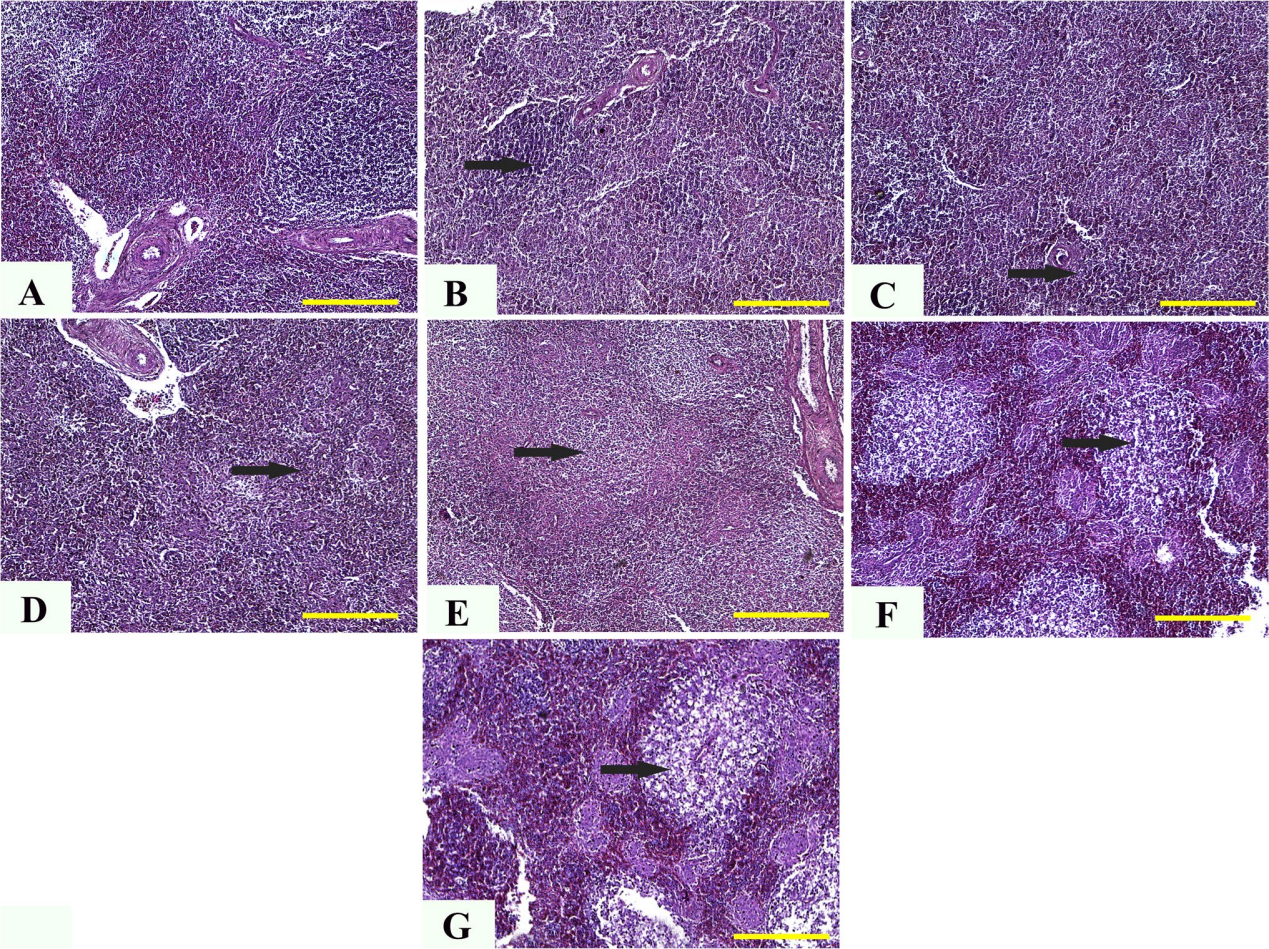
Representative photomicrographs from the spleen of broilers treated with different concentrations of ZnO NPs for 35 days: H&E staining. The control group (**A**), broilers treated with 5 ppm of ZnO NPs (**B**), broilers treated with 10 ppm of ZnO NPs (**C**), broilers treated with 20 ppm of ZnO NPs (**D**), broilers treated with 40 ppm of ZnO NPs (**E**), broilers treated with 60 ppm of ZnO NPs (**F**), and broilers treated with 80 ppm of ZnO NPs (**G**) showing normal histological structure of lymphoid follicles, white and red spleen pulps (**A**), multifocal lymphoid depletion and reduction of lymphoid follicles size (arrows) (**B**-**D**), complete absence of lymphoid follicles (arrow) (**E**), marked depletion and necrosis of the entire white pulps (**F**, **G**). ZnO NPs, zinc nanoparticle; H&E, hematoxylin and eosin. Scale bars = 100 μm (× 10)

Splenic tissues from the control group revealed the normal histological structure of lymphoid follicles and white and red pulps (Fig. 5A). In addition, chickens given the smaller doses of ZnO NPs (5, 10, and 20 mg) showed multifocal lymphoid depletion and reduction of lymphoid follicle size (Fig. 5B and C). However, chickens given 40–80 mg ZnO NPs demonstrated a complete absence of lymphoid follicles (Fig. 5D), with marked depletion and necrosis of the entire white pulps (Fig. 5E and F).

#### Thymus

**Fig. 6:**
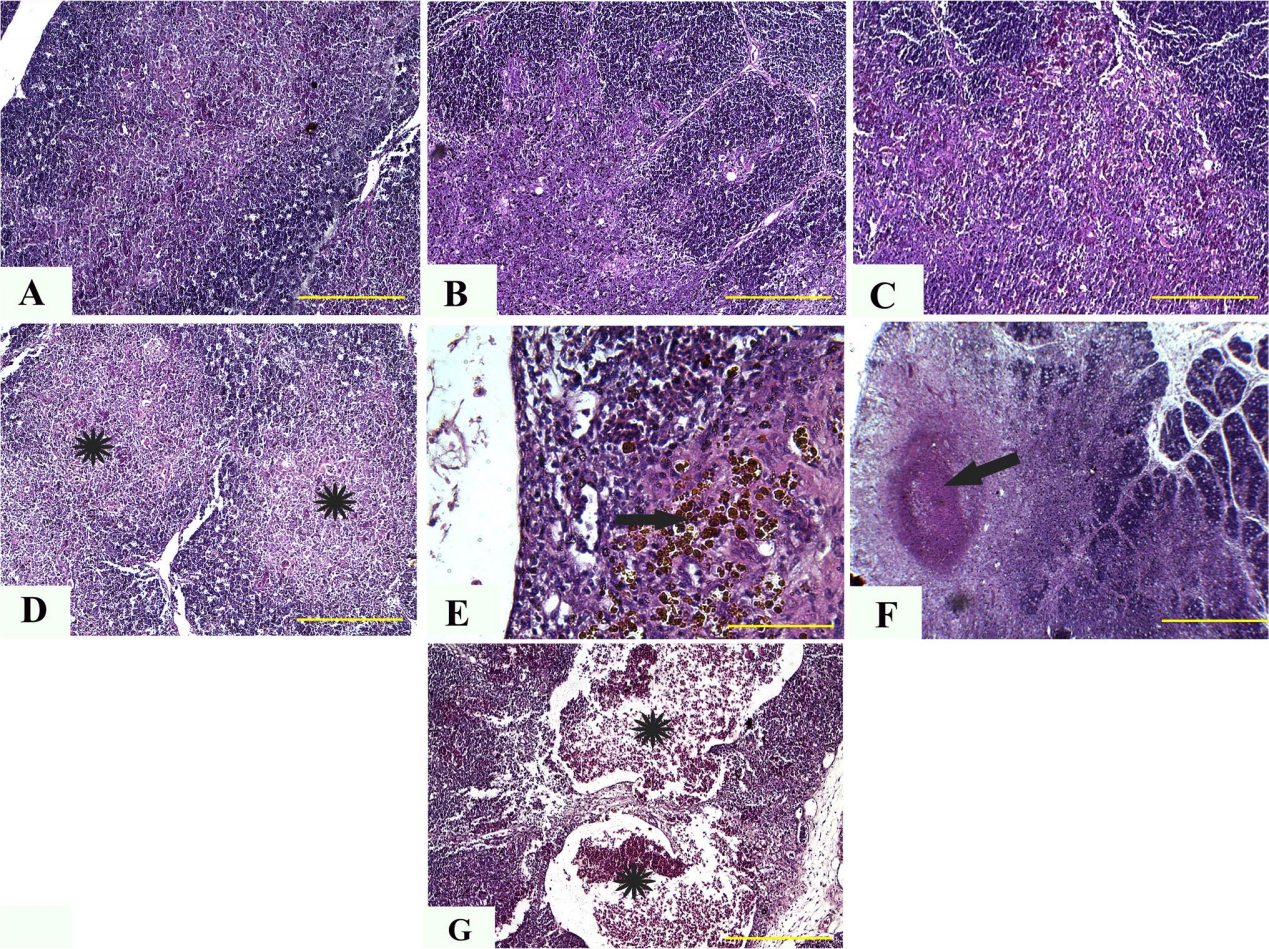
Representative photomicrographs from the thymus of broilers treated with different concentrations of ZnO NPs for 35 days: H&E staining. The control group (**A**), broilers treated with 5 ppm of ZnO NPs (**B**), broilers treated with 10 ppm of ZnO NPs (**C**), broilers treated with 20 ppm of ZnO NPs (**D**), broilers treated with 40 ppm of ZnO NPs (**E**), broilers treated with 60 ppm of ZnO NPs (**F**), and broilers treated with 80 ppm of ZnO NPs (**G**) showing normal architecture and intensity of cortical and medullary thymocytes (**A**–**C**), marked reduction of cortical and medullary thymocytes (stars) (**D**), severe reduction of medullary and cortical basophilic thymocytes and accumulation of hemosiderin-laden macrophages (arrow) (**E**), large infarct area within medullary tissues (arrow) (**F**), and perifollicular edema with severe congestion (stars) and hemorrhage (**G**). ZnO NPs, zinc nanoparticle; H&E, hematoxylin and eosin

Examination of thymus tissues from control and treated chickens (5 and 10 mg) revealed normal architecture and intensity of cortical and medullary thymocytes with prominent corticomedullary junctions (Fig. 6A–C). However, chicks given 20 mg ZnO NPs showed a marked reduction of cortical and medullary thymocytes (Fig. 6D). However, thymus tissues from chicken given 40, 60, and 80 mg ZnO NPs showed a severe reduction of medullary and cortical basophilic thymocytes and accumulation of hemosiderin-laden macrophages (Fig. 6E), large infarct area within medullary tissues (Fig. 6F), and perifollicular edema with severe congestion and hemorrhage (Fig. 6G).

### IL-1β and TNF-α mRNA expression in muscle tissues

**Fig. 7:**
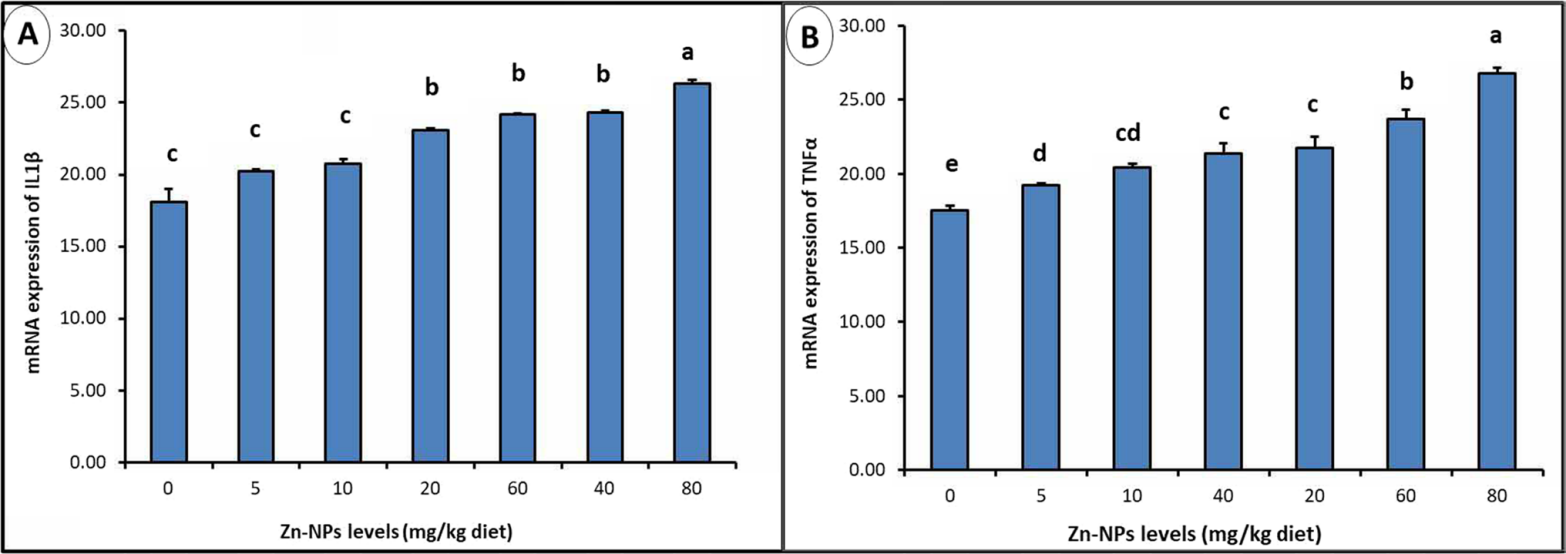
Impact of ZnO NPs on mRNA expression of IL1β and TNF-α in muscle tissue. Groups having several letters are significantly different at (*P* < *0.05*). ZnO NPs; ILβ1 and TNF-α

IL-1β mRNA expression was investigated (Fig. 7A). The degree of IL-1β expression in muscle tissues was significantly higher (*P* > *0.05)* in chickens given 20, 40, 60, and 80 mg ZnO NPs than the other treated and control birds in a dose-dependent manner. In contrast, a nonsignificant difference (*P* ≤ *0.05*) was detected in chicken given 5 and 10 mg ZnO NPs compared to the control group. In addition, compared to the control group, TNF-α mRNA expression in muscle tissues of chickens given ZnO NPs at all dose levels revealed a significantly (*P* ≤ *0.05*) dose-dependent upregulation (Fig. 7B).

#### Assessment of ultrastructural morphology of pectoral muscles of chickens

**Fig. 8:**
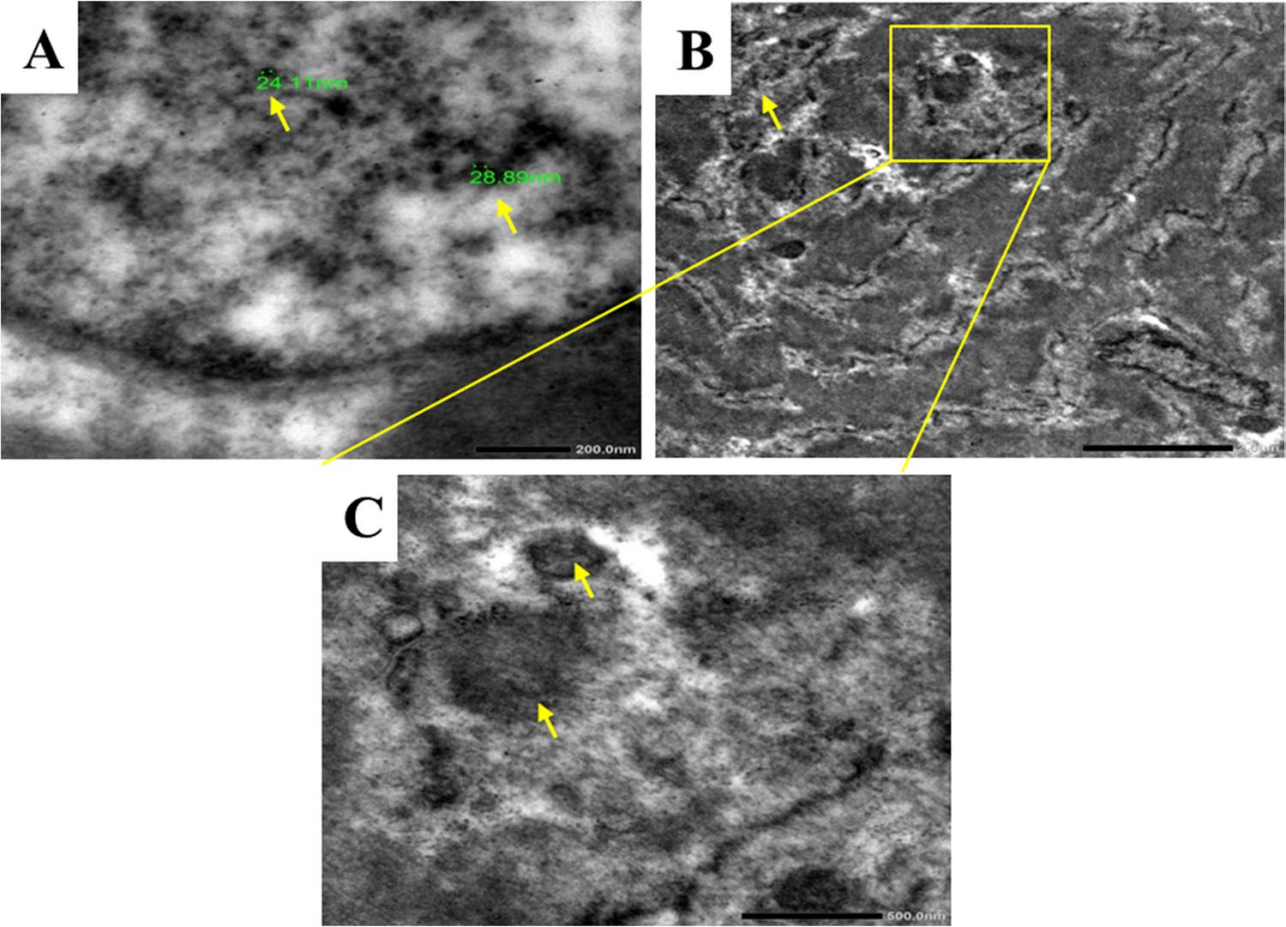
Showing the ultrastructure morphology of muscle tissue from Zn NP-treated broilers. **A** An irregular nucleus with disintegrated nuclear chromatin and aggregations of Zn NPs deposits within the nuclear chromatin. **B** Numerous areas of degenerated fibers and mild cytoplasmic vacuolization (arrows). **C** Fragmented mitochondrial cristae and aggregations of Zn NP deposits

Compared to the control group, TEM was used to validate the existence or absence of ZnO NPs and localize their presence in the pectoral muscles of chickens. The nucleus, nuclear envelope, and spherical or ovoid-shaped mitochondria with well-developed cristae, filaments, and Z-bands were present in control muscle cells. In contrast, treated muscles had an uneven nucleus and irregular nuclear envelope, fragmented nuclear chromatin, and aggregation of ZnO NP deposits within the nuclear chromatin (Fig. 8A). Degenerated fibers, minor cytoplasmic vacuolization, fractured mitochondrial cristae, and aggregation of ZnO NPs were also found within the mitochondrial cristae and lysosome internal membranes (Fig. 8B and C).

## Discussion

This study showed nonsignificant effects for live weight, gain, growth rate percentage, PI, feed consumption, and FCR between treated and control birds. Similarly, in Asheer et al. (2018), no significant differences were observed among 0.0, 25%, 50%, 75%, and 100% of ZnO NP treatments on the final live body weight, cumulative feed intake, and cumulative FCR at the sixth week of broiler age. However, these results indicated a numerical increase in growth performance parameters and agreed with those in El-Katcha et al. (2017b), which showed that 60, 30, and 45 ppm ZnO NPs/kg in broilers diet improved the growth performance and feed efficiency parameters. In contrast with our data, the authors in Hafez et al. (2017) have stated that a diet containing 40 and/or 80 mg/kg ZnO NPs is a significant Zn source for chicken, with beneficial effects on performance. Additionally, diets with ZnO at 20 or 40 mg/kg (Fathi et al. 2016) or 60 mg/kg (Hussan et al. 2022; Pathak et al. 2016) improved the growth performance of broiler chicks and reduced mortality due to ascites. Moreover, researchers (Ibrahim et al. 2017) have found that ZnO NPs improved Zn retention, enzyme antioxidant activity, and metabolism of broiler chickens, resulting in better performance. In contrast, in Mohammadi et al. (2015), it has been revealed that ZnO NP sulfate (80 mg/kg diet) decreased BWG of broilers from 1 to 42 days. The conflicting results of different studies could be related to the differences in the physical and chemical features of the different sources of Zn. Many studies revealed that the improvement in broiler growth performance parameters when ZnO NP supplementation is added in broiler diet might be due to zinc polysaccharide uptake related to organic minerals, which are a good vehicle to supply broilers with more minerals without increasing dietary mineral levels (Abdallah et al. 2009). Moreover, another reason is that the zinc nanoparticle size has a faster diffusion through GIT membrane, resulting in higher uptake of zinc nanoparticles in the gastrointestinal tract. The differences between this study and other research papers may be attributed to differences in concentration levels, breed, and environment and management procedures.

Among the hematological and immunological variables, WBC and RBC counts, PCV, lymphocytes (%), heterophils (%), lymphocytes/heterophils ratio, and monocytes were significantly (*P* ≤ *0.05*) influenced by ZnO NP supplementation. However, Hb concentration, basophils (%), and eosinophils (%) were not significantly detected in the different treatments. These results indicated that the use of ZnO NPs has no detrimental effect on the hematological parameters of broilers. On the contrary, in Salama et al. (2003), Zn at higher concentrations tends to inhibit copper and iron absorption that are required for WBC and RBC maturation and proliferation; therefore, further investigation concerning blood parameters on different ZnO NP is warranted. In partial agreement with these findings, in Abed and Ezzat (2021), nonsignificant differences in PCV and Hb concentrations in the blood were found when adding ZnO NPs to broilers feed at 21 and 42 days.

In this study, the antibody titer against the NDV showed a nonsignificant difference between the control and treated groups. Similarly, in Abed and Ezzat (2021) and Khalifa et al. (2021), no significant differences among different groups given nano- and ZnO NPs for NDV disease were found (*P* ≤ *0.05*). In contrast, the authors in Khajarern et al. (2002) have indicated that high levels of Zn supplementation (75 vs. 175 mg/kg) have led to a high antibody titer for NDV disease and IB. In Kim et al. (2014), differently sized and charged ZnO NPs would cause in vitro and in vivo immunotoxicity, which is considered a naturally immunosuppressant. On the other hand, researchers in Sahoo et al. (2014) assumed that dietary ZnO NPs might have elicited a better immune response even at lower physiological limits.

This study revealed a significant reduction in IgM and IgG in broilers given high levels of ZnO NPs (60 and 80 ppm/kg diet). In partial agreement with this finding, the authors in Hafez et al. (2020) have shown that birds fed diets supplemented with ZnO NPs had a significant increase (*P* ≤ *0.05*) in IgY, total lymphocyte counts, and macrophages compared with the control. However, this highly significant response was observed in birds given the 5 ppm ZnO NPs/kg diet rather than the 80 ppm ZnO NPs/kg diet. On the contrary, the authors in Sahoo et al. (2014) found that when 15 ppm organic zinc and 0.06 ppm nano-zinc were added to the basal diet, the antibody titer and immune organ response were increased, thus improving the immune status of the birds. Another study (Kidd et al. 1996) discussed the immunity-enhancing role of Zn as it can increase the thymocytes and peripheral T cells count and enhance the interferon and interleukin-2 production. Therefore, increasing the bioavailability of zinc, as in the ZnO NPs, might promote immune responses via induction of extra thymulin activity, which subsequently enhances the maturation of T lymphocytes and the activation of B lymphocytes by T helper cells (Abedini et al. 2018).

In this study, significant increases in PA and PI were observed in groups fed ZnO NPs, with a higher significance in the group given the 20 ppm ZnO NPs/kg diet than that given the 80 ppm ZnO NPs/kg diet compared to the control. These results agree with those in Sahoo et al. (2014), which indicated that 15 ppm Zn in organic form and 0.06 ppm Zn NPs increased the antibody titer of the birds. In contrast, Zn from an inorganic source had not improved the birds’ immunity status (Abedini et al. 2018, Moghaddam and Jahanian 2009, Sahoo et al. 2014). These results were consistent with those in El-Katcha et al. (2017b), which showed an enhancement in the PI of broilers fed with 45 ppm ZnO NPs. These findings were also parallel to those in Chand et al. (2021), Sahoo et al. (2014), and Swain et al. (2015), which indicated that compared to control, Zn NPs seem to be more bioavailable even at lesser levels, IL-2 was significantly secreted, and cell-mediated immune response was better (Prasad et al. 2002). The increase in immune response parameters due to Zn NP uptake may be attributed to the enhanced maturation of T lymphocyte and activation of B lymphocytes by T helper cells (Hudson et al. 2004). Moreover, the authors in O'Dell (1992) concluded that the immune system is dependent on the functions of cellular metabolism. Zinc is ubiquitous in cellular metabolism and functions both structurally and catalytically in metalloenzymes. However, this description did not show matches with our histopathologic results of immune organs, where dose-dependent lymphoid depletion was discovered in ZnO NP-treated chicks. Further confirmatory studies are needed in this point to uncover the possible causes of this difference.

In this study, a significant dose-dependent increase in triglycerides and nonsignificant changes in LDL and cholesterol levels were reported in treated groups in equivalence to the control group. These obtained data were in partial agreement with those in Abed and Ezzat (2021) and Hussan et al. (2022), where no significant effect was found among the treatments in total cholesterol at 21 and 42 days of age and triglyceride concentration at 21 days of age, whereas triglyceride concentration was significantly affected at 42 days of age. In addition, in Zaghari et al. (2013), an increase in triglyceride was reported when birds were fed with Zn, in turn affecting lipid metabolism (Aksu et al. 2010). In contrast with these findings, in Ahmadi et al. (2013), a reduction in triglycerides and LDL was noticed when 60 and 90 mg/kg ZnO NPs were added to birds’ diets. Additionally, in Zaghari et al. (2013), a reduction in triglyceride and an increase in LDL in serum were observed in birds fed a diet supplemented with 100 mg ZnO. In agreement with these findings, other studies Sarvari et al. (2015) and Hussan et al. (2022) and Radi et al. (2021) showed that ZnO NP supplementation did not influence cholesterol and serum protein concentrations at 42 days of age.

Oxidative stress is primarily uncovered via the modulation of the antioxidant enzyme and the downregulation of the nonenzymatic antioxidants. In this study, TAC and CAT were significantly (*P* ≤ *0.05*) reduced with increased ZnO NP dose in the diet compared with the control. Furthermore, MDA concentrations were significantly (P ≤ 0.01) increased by increasing ZnO NPs in the diets. Contrary to these results, in Alam et al. (2018) and Zhao et al. (2014), CAT activity was increased and maintained by 20 ppm of ZnO NPs compared to 60 ppm ZnO (Eskandani et al. 2021; Zhao et al. 2014). Furthermore, in Fathi et al. (2016), 20 mg/kg ZnO NPs significantly reduced MDA compared to the control. Other authors have discussed their results regarding the upregulation of Nrf2, which may be a major mechanism controlling Zn antioxidant action (Abdel-Daim et al. 2019; Cortese et al. 2008; Eskandani et al. 2021) or the competition of Zn with copper and iron for binding to the cell membrane and decreasing the free radicals production (Tate et al. 1999). The differences between this study and previous research may be due to differences in dose, breed, and environment and management procedures.

In a more recent study (Bartlett and Smith 2003), blood zinc levels were not affected by zinc quantity in the diet of birds subjected to heat stress. Additionally, similar results were stated (Hussan et al. 2022; Sarvari et al. 2015) regarding broiler growth and various levels of supplemented Zn. Similarly, in Feng et al. (2010a), no positive effect on immune organ weights was found. On the contrary, in Khah et al. (2015), significantly (*P* ≤ *0.05*) enhanced dressing percentage and carcass and breast weight with ZnO NPs were reported. Moreover, in Sahoo et al. (2014), an increase in immune organ weights with ZnO NPs in broilers was observed.

In our study, the histopathologic examination of different tissues supports the chemical, immune-related, and oxidative findings, where there was dose-dependent damage in the liver, kidney, spleen, bursa, and thymus, which may be correlated to the dose-dependent oxidative damage described above. Limited studies are available regarding the detailed description of histopathologic changes associated with Zn (Wight et al. 1986) or ZnO NPs (El-Katcha et al. 2017a; Radi et al. 2021). At the same time, no previous studies are available regarding the description of such pathologic lesions in lymphoid organs. In the present study, the predominant pathological lesions could also be attributed to their solubility, increasing intracellular Zn2 + (Saman et al. 2013). In previous studies, the authors reported nanoparticle-related inflammatory reactions in different tissues, particularly the lymph nodes. Moreover, in Watson et al. (2015), the inhibition of Kupffer cell phagosomal motility by ZnO NPs with consequent hepatic damage was reported. In addition, the ultrastructure examination of muscles tissues in this study revealed several dose-dependent lesions such as the irregular nucleus, disintegrated nuclear chromatin, numerous areas of degenerated fibers and mild cytoplasmic vacuolization, and fragmented mitochondrial cristae. These lesions could be attributed to oxidative stress and the upregulations in gene expression of IL1α and TNF-α. This correlated increment in ROS production and inflammatory cytokines can diminish the mitochondrial function within cells (Hussain et al. 2005; Khalifa et al. 2021; Xia et al. 2006), leading to moderate to severe damage in the internal morphology of different organs.

## Conclusion

Based on the obtained findings, ZnO NPs could be practically used in broiler diet at doses of 5 and 10 ppm/kg diet as an alternative of high-dose zinc oxide as these doses showed the most favorable effects and less toxic effects on the immune status of birds, followed by 20 ppm. Moreover, the histopathological examination of internal organs revealed dose-dependent morphological and structural changes for the kidney, liver, and lymphoid organs (bursa, spleen, and thymus). Consequently, more than 10 ppm ZnO NPs/kg diet is not recommended as this might induce harmful effects on the immune status and histologic structure of immune organs. Further studies are needed to properly detect the possible toxic effects and mechanism/s of ZnO NPs.

## Data Availability

The data used to support the findings of this study are included within the article, and the coding of the data is available from the corresponding author upon reasonable request.
